# Correlation Between Number of Retrieved Oocytes and Pregnancy Rate After *In Vitro* Fertilization/IntraCytoplasmic Sperm Infection

**DOI:** 10.1100/tsw.2006.145

**Published:** 2006-06-21

**Authors:** Tanya Timeva, Tanya Milachich, Irena Antonova, Tanya Arabaji, Atanas Shterev, Hatim A. Omar

**Affiliations:** ^1^ IVF Unit, Ob/Gyn Hospital “Dr. Shterev”, Sofia, Bulgaria; ^2^ Section of Adolescent Medicine, University of Kentucky, Lexington, USA

**Keywords:** assisted reproductive technology, oocyte, ovarian stimulation, IVF, ICSI, pregnancy rate, Bulgaria

## Abstract

The implementation of safe and maximally effective ovarian stimulation is a major aim for *in vitro* fertilization (IVF) teams. The goal of controlled ovarian hyperstimulation (COH) is to supply enough oocytes with normal maturation to insure the consequent biological procedures. A variety of different stimulation protocols have been suggested and an individual selection of the correct stimulation protocol is mandatory. The aim of the present study is to evaluate the correlation between number of retrieved oocytes and clinical pregnancy rate (CPR) after IVF or intracytoplasmic sperm injection (ICSI) procedures. We reviewed 1017 cycles in a total of 975 patients. The study results clearly demonstrate that the aspiration of less than 5 oocytes significantly reduced pregnancy rate. The aspiration of a large number of oocytes (>15) does not lead to an increase of the treatment effect and, at the same time, increases the risk of ovarian hyperstimulation syndrome. The major goal is to obtain 5—15 oocytes as a “gold standard”, connected to optimal pregnancy rate after assisted reproduction (ART).

## INTRODUCTION

Numerous studies have investigated the factors that affect the outcome of *in vitro* fertilization (IVF) treatment. The assisted reproduction cycles outcome is dependent on several factors: female age[1], etiology of sterility[2,3], previous ovarian surgery[4], embryo morphology and cleavage rates[5], but also the technique of embryo transfer[6,7]. It is well established that age is the major determining factor and none of the ways of reversing the effects of age on the outcome of IVF treatment have been proven to be effective. One of the most important steps in assisted reproductive technology (ART) is to obtain adequate ovarian response to gonadotropins during controlled ovarian hyperstimulation (COH). The number of mature follicles on the day of human chorionic gonadotrophin (HCG) administration (noted on ultrasound[8,9]), peak serum estradiol concentration, number of oocytes retrieved[10], or combination of these parameters[11,12] have been used by most authors as criteria to define ovarian response.

The aim of the present study was to evaluate the correlation between number of retrieved oocytes and clinical pregnancy rate (CPR) after IVF/ICSI (intracytoplasmic sperm injection) procedures. The tasks we assigned were to compare the CPR after IVF/ICSI in the different groups of patients, dependent on the number of retrieved oocytes after pickup.

## METHODS

Data of IVF/ICSI cycles performed from February 1999 to December 2005 in the reproductive unit were reviewed. The retrospective analysis included a total of 1017 cycles in 975 female patients at mean age 32.13 years (SD ± 4.78) and the average duration of couple infertility is 5 years and 4 months (SD ± 3.09).

We used a long protocol with gonadotropin-releasing hormone (GnRH) agonists in 68.14% (693/1017), GnRH antagonists protocol in 30.19% (307/1017), and a short protocol in 1.67% (17/1017). The mean duration of stimulation was 13 days. HCG was given when follicles were ≥18 mm. A single dose of 10,000 IU of HCG was administered and oocyte recovery was timed for 36 h later.

Patients were divided into five groups depending on the number of retrieved oocytes after pickup:

| | | |
|---|---|---|
| *Group I:* | 15 (1.47%) | 0 oocytes |
| *Group II:* | 408 (40.12%) | 1–5 oocytes |
| *Group III:* | 412 (40.51%) | 6–10 oocytes |
| *Group IV:* | 158 (15.54%) | 11–15 oocytes |
| *Group V:* | 24 (2.36%) | Over 15 oocytes |

### Statistical Analysis

Comparison between two outcomes (pregnant and nonpregnant) was carried out by the nonparametric Mann-Whitney test. A *p*-value (two-tailed) < 0.05 was considered statistically significant.

## RESULTS

The oocyte pickup was followed by IVF and ICSI procedures, respectively, in 517 (50.84%) and 412 (40.51%); a combination of both methods (IVF + ICSI) in 76 (7.47%); and PESA/ICSI in 12 (1.18%) cases (Fig. 1).

In 1.47% (15/1017) of the stimulated patients, no viable oocytes were retrieved after pickup. In 4.62% (47/1017), because of lack of fertilization, embryo transfer (ET) was not performed. It means that in 6.09% (62/1017) of all patients who underwent COH, the procedures were not completed. Fresh ET was successfully completed in 955 out of 1017 patients (93.91%). We observed the following CPR/ET per groups: Group I – 0%; Group II – 13.82%; Group III – 28.22%; Group IV – 34.81%, and Group V – 25% (Table 1).

We observed better results – CPR 28.22% and 34.81%, respectively, in Groups III and IV – which were statistically higher then the CPR in the other groups. We considered as clinical pregnancy only the cases where a fetal heart beat was established. In Group V, the CPR was lower and, at the same time, these patients were at risk to develop hyperstimulation syndrome. The data of Group II, women with low ovarian response to COH, were analyzed in detail and the results are shown in Table 2.

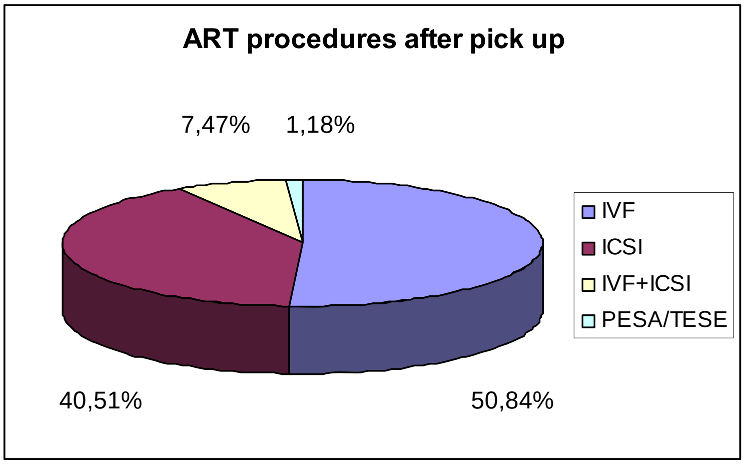

**FIGURE 1**. Distribution of procedures, following oocyte retrieval.

**TABLE 1**
**Clinical Pregnancy Rate After IVF/ICSI, According to the Number of Retrieved Oocytes**

| Group | No. of Oocytes | Cycles No. (%) | Women with ET No. (%) | CPR/ET No. (%) |
|---|---|---|---|---|
| I | 0 | 15 (1.47%) | 0 (0%) | — |
| II | 1–5 | 408 (40.12%) | 369 (90.44%) | 51/369 (13.82%) |
| III | 6–10 | 412 (40.51%) | 404 (98.06%) | 114/404 (28.22%)* |
| IV | 11–15 | 158 (15.54%) | 158 (100%) | 55/158 (34.81%)* |
| V | >15 | 24 (2.36%) | 24 (100%) | 6/24 (25%) |

*$p < 0.05$.

**TABLE 2**
**Pregnancy Rates in Low-Responding Women (Group II: 1–5 Oocytes)**

| No. of Oocytes | No. of Cases | Women without ET No. (%) | CPR/ET No. (%) |
|---|---|---|---|
| 1 | 27 | 11 (40.74%) | 0/16 (0 %) |
| 2 | 74 | 11 (14.86%) | 8/63 (12.70%) |
| 3 | 103 | 7 (6.80%) | 9/96 (9.38%) |
| 4 | 104 | 6 (5.77%) | 12/98 (12.25%) |
| 5 | 100 | 4 (4.0%) | 22/96 (22.92%)* |

*$p < 0.05$.

These results showed a statistically significant ($p < 0.05$) increase in CPR when a minimum of five oocytes was retrieved. No pregnancy occurred in the patients with only one aspirated oocyte, but it was totally different from the results in spontaneous cycles, which were not included in this study.

## DISCUSSION

One aspect that all IVF teams agree on is the usefulness of using follicular stimulation protocols to obtain more oocytes. Our data confirmed that a low development of follicles, less than 5, following ovarian stimulation was usually associated with poor outcome (CPR = 12.25%), related to the low number of retrieved oocytes and respectively transferred embryos. High cycle cancellation rate (9.56% women without ET, in Group II) might be due to an undetected fertilization of oocyte with poor quality or uncleaved embryos. This hypothesis is supported by other authors who have reported plenty of aneuploidies in early embryos, obtained from low responders[13,14]. Statistically significant increased pregnancy rate in the groups of patients with 5 or more oocytes is related to a chance for choosing the best embryos for transfer, a possibility for blastocyst ET. The optimal results were reached in Groups III and IV. We observed a slight decrease in the pregnancy rate (CPR = 25%) when more than 15 oocytes were retrieved, and it might be due to changes related to ovarian hyperstimulation syndrome and the negative effect of high estradiol level on implantation[15].

In conclusion, this study showed that the number of retrieved oocytes could be used as a prognostic criterion for the treatment outcome as there was a correlation between this factor and the CPR. The aspiration of 4 or less oocytes was related to significant reduction of success rate. At the same time, production of a large number of oocytes (>15) did not lead to an increase of the treatment effect. We propose that a "golden standard" for optimal results of treatment is retrieved of 5 to 15 oocytes.

## BIOSKETCHES

**Tanya Timeva, MD,** Resident in Obstetrics and Gynecology and Reproductive Medicine, Medical Director of Obstetrics and Gynecology Hospital “Dr. Shterev”, Sofia. Dr. Timeva is a member of BASRZ and ESHRE. She is leader of the project of Ministry of Education in Bulgaria. E-mail: ttimeva@yahoo.com

**Tanya Milachich,** Embryologist in IVF unit, Ob/Gyn Hospital “Dr. Shterev”, Sofia. E-mail: mcrz@mail.bg

**Irena Antonova,** Resident Embryology, Institute of Sterility and ART, Sofia. E-mail: mcrz@mail.bg

**Tanya Arabaji,** Undergraduate student, Medical University, Sofia. E-mail: mcrz@mail.bg

**Atanas Shterev, MD, PhD,** Associate Professor of Obstetrics and Gynecology, Medical University, Sofia. He has numerous publications in the area of assisted reproduction. E-mail: mcrz@mail.bg

**Hatim A. Omar, MD,** Professor of Pediatrics and Obstetrics and Gynecology and Director of the Section of Adolescent Medicine, Department of Pediatrics, University of Kentucky, Lexington. Dr. Omar has completed residency training in obstetrics and gynecology as well as pediatrics. He has also completed fellowships in vascular physiology and adolescent medicine. He is the recipient of the Commonwealth of Kentucky Governor’s Award for Community Service and Volunteerism and is well known internationally with numerous publications in child health, pediatrics, adolescent medicine, pediatric and adolescent gynecology. E-mail: haomar2@uky.edu; Website: http://www.ukhealthcare.uky.edu/kch/physicians/omar.htm
